# Occupational pesticide exposure and the risk of death in patients with Parkinson’s disease: an observational study in southern Brazil

**DOI:** 10.1186/s12940-020-00624-8

**Published:** 2020-06-17

**Authors:** Márcio Schneider Medeiros, Sumanth P. Reddy, Mariana P. Socal, Artur Francisco Schumacher-Schuh, Carlos Roberto Mello Rieder

**Affiliations:** 1 Serviço de Neurologia, Hospital de Clínicas de Porto Alegre, Universidade Federal do Rio Grande do Sul, R. Ramiro Barcelos, 2350 - Santa Cecilia, Porto Alegre, RS 90040-060 Brazil; 2 Department of Global Health, University of Texas Southwestern Medical Center, Dallas, TX USA; 3 Department of Health Policy and Management, Johns Hopkins Bloomberg School of Public Health, Baltimore, MD USA; 4 Departamento de Farmacologia, Universidade Federal do Rio Grande do Sul, Porto Alegre, RS Brazil; 5 Departamento de Neurologia, Universidade Federal de Ciências da Saúde de Porto Alegre, Porto Alegre, RS Brazil

**Keywords:** Pesticides, Parkinson, Mortality, Occupational exposure, Environmental exposure

## Abstract

**Background:**

Multiple studies have suggested that various pesticides are associated with a higher risk of developing Parkinson’s disease (PD) and may influence the progression of the disease. However, the evidence regarding the impact of pesticide exposure on mortality among patients with PD is equivocal. This study examines whether pesticide exposure influences the risk of mortality among patients with PD in Southern Brazil.

**Methods:**

A total of 150 patients with idiopathic PD were enrolled from 2008 to 2013 and followed until 2019. In addition to undergoing a detailed neurologic evaluation, patients completed surveys regarding socioeconomic status and environmental exposures.

**Results:**

Twenty patients (13.3%) reported a history of occupational pesticide exposure with a median duration of exposure of 10 years (mean = 13.1, SD = 11.2). Patients with a history of occupational pesticide exposure had higher UPDRS-III scores, though there were no significant differences in regards to age, sex, disease duration, Charlson Comorbidity Index, and age at symptom onset. Patients with occupational pesticide exposure were more than twice as likely to die than their unexposed PD counterparts (HR = 2.32, 95% CI [1.15, 4.66], *p* = 0.018). Occupational pesticide exposure was also a significant predictor of death in a cox-proportional hazards model which included smoking and caffeine intake history (HR = 2.23, 95% CI [1.09, 4.59], *p* = 0.03)) and another which included several measures of socioeconomic status (HR = 3.91, 95% CI [1.32, 11.58], *p* = 0.01).

**Conclusion:**

In this prospective cohort study, we found an increased all-cause mortality risk in PD patients with occupational exposure to pesticides. More studies are needed to further analyze this topic with longer follow-up periods, more detailed exposure information, and more specific causes of mortality.

## Background

The etiology of Parkinson’s disease (PD) is a complex interplay of environmental and genetic factors. Although several genes have been implicated as monogenic causes of the disease, these genetic mutations are only responsible for approximately 10% of cases [1]. The remaining 90% of the cases are idiopathic, and different environmental exposures have been implicated as either protective factors (such as tobacco smoking and caffeine intake) or risk factors (such as heavy metals and pesticides exposure) [1].

The term pesticides refers to herbicides, insecticides, rodenticides, fungicides, and other chemical agents that eliminate unwanted organisms [2]. Paraquat, a herbicide with molecular similarities to MPP+ (1-methyl-4-phenylpyridinium, a metabolite of the neurotoxin MPTP (1-methyl-4-phenyl-1,2,3,6-tetrahydropyridine)), is among the earliest and most well studied pesticides linked to an increased risk of developing PD. In addition to preferentially damaging dopaminergic neurons, these agents share several common mechanisms of action including increasing neuronal oxidating stress, damaging mitochondrial complex I, and impairing the ubiquitin-proteasome system [3, 4].

A recent meta-analysis of several case-control studies found that paraquat exposure was associated with a 1.64 times increased risk of developing PD [5]. In addition to paraquat, subsequent studies, systematic reviews, and meta-analyses have suggested that a wide range of pesticides (including rotenone, maneb, organochlorines, and organophosphates) are associated with an increased risk of PD [6]. In recent meta-analyses, the risk of developing PD is 1.28–1.94 times higher among those with unspecified pesticide exposure [7–12]. However, this association may be largely driven by insecticides and herbicides rather than fungicides and rodenticides [7].

In addition to increased risk, data suggests that pesticide exposure is associated with earlier onset of symptoms [13], with premature death in PD patients who are exposed to glyphosate [14] and with an influence in the progression of motor, cognitive and psychiatric symptoms [3]. Considering this data, pesticides exposure may contribute to all stages of PD.

Patients with PD typically are at an increased risk of mortality compared to unaffected controls, with an overall mortality ratio of 1.52 [15]. Furthermore, studies that investigated PD mortality found increased mortality rates for individuals living in areas with higher levels of pesticide use compared to their controls [14, 16, 17].

The role of pesticides exposure in the development and progression of PD is particularly important to understand in the context of Brazil and other low- and middle-income countries (LMIC). In recent years, the Brazilian government has approved swath of new pesticides, many of which contain substances that are illegal in the European Union. A record 450 new agrochemicals were approved in 2018, and based on data from the early months of this 2019, the new administration was on track to approve 480 new products within this 2019 alone [18].

Considering that the use of these substances is projected to increase in the coming years, the objective of this prospective cohort study was to determine if pesticide exposure is associated with an increased risk of mortality among patients with PD in Southern Brazil, when accounting for socioeconomic status, nicotine exposure, and caffeine exposure.

## Methods

The data for this study is part of a larger cohort of 233 patients with idiopathic PD (as defined by the UK Parkinson Disease Brain Bank Diagnostic Criteria) who are followed at the Movement Disorders Clinic at Hospital de Clínicas de Porto Alegre (HCPA) and previously described [19]. Patients were enrolled consecutively from 2008 to 2013 and followed until 2019. The clinic is part of a tertiary health care system in Porto Alegre, a city in southern Brazil with a population of approximately 1.4 million. Ethics approval for this study was provided by Comitê de Ética em Pesquisa from HCPA. All patients or their next-of-kin provided written informed consent for participation in this study.

A subset of 150 patients completed questionnaires regarding environmental exposure history, and 126 patients completed an additional questionnaire regarding socioeconomic history during their period of follow up. This data was collected by the researchers during clinic visits, often with the help of a family member. The environmental exposure survey included information regarding occurrence and duration of occupational pesticide exposure (yes/no question and approximate number of years of exposure), smoking history, alcohol use history, and caffeine intake. Since pesticide exposure is difficult to accurately quantify among the general population, we asked patients to report pesticide exposure in occupational settings, such as agriculture, landscaping, or pesticide production. Household use of insecticides, herbicides used in the garden and products for house pets were excluded. The socioeconomic survey included information regarding employment history, average historical monthly income (when employed), insurance status, race, and education level. In addition to the survey information, each patient underwent neurologic evaluation (UPDRS and Hoehn & Yahr) by a neurologist trained in movement disorders at the time of enrollment. Charlson Comorbidity Index was used to assess the comorbidities of patients at baseline using information from the patients´ medical records.

All statistical analysis was performed using R-Studio (Version 3.5.2). To determine survival time, clinic and hospital data was searched to determine whether patients were alive as of January 1, 2019. For patients who were lost to follow up, national mortality databases were searched to identify confirmed deaths [20]. Patients who were lost to follow up, and without a confirmed date of death were right censored in the mortality analysis at the last known date of hospital admission or clinic follow up. For all survival analyses, the data was left-truncated and right censored, with age as the time scale, starting at study enrollment. This was selected rather than time-in-study due to ease of interpretability [21, 22]. Age was also viewed as a more objective measure than disease duration, which was derived from the patients’ best guess of their symptom onset.

The primary analysis was a survival analysis testing (Kaplan-Meier curve with log-rank test; unadjusted hazard ratio) to compare PD patients who were exposed to occupational pesticides to those who were not. The secondary analyses sought to understand the impact of confounding of other environmental exposures and socioeconomic factors on the survival difference between the two cohorts. Because the number of individuals exposed to occupational pesticides in our sample was low, we examined the association between occupational exposure to pesticides and mortality adjusting for confounders through two separate multivariable cox proportional hazards models. The first cox proportional hazards model was adjusted to disease duration at enrollment, demographic, and exposure-related factors and included any history of smoking, history of caffeine intake (80 mg or more per day, for at least 10 years), sex, and occupational pesticide exposure. The second cox proportional hazards model was adjusted to disease duration at enrollment, socio-economic factors and included sex, low historical income (< R$500/month), predominately agricultural employment, low education, private insurance coverage. Private health insurance coverage was used as a proxy of current wealth, since it is typically prohibitively expensive for the average Brazilian. In order to test the proportional hazards assumption for the survival analyses, the Schoenfield Residuals Test was applied to each model.

In order to test for a dose-response relationship between occupational pesticide exposure and risk of mortality, we performed an additional post-hoc cox proportional hazards model. Patients were divided into low exposure (< 10 years) and high exposure cohorts (≥10 years) based on the median duration of occupational pesticide exposure in this model, using the no exposure group as reference.

## Results

Of the 150 patients in this prospective cohort, 20 (13.3%) reported a history of occupational pesticide exposure. Females comprised 54% of the overall cohort. On average, patients were 64.4 years old (SD = 11.7) with 7.9 years of motor symptoms (SD = 5.2) at the time of study enrollment. A total of 28.7% of patients had symptom onset prior to the age of 50 years. Twenty-two (14.7%) patients were lost to follow-up, after an average follow-up period of 8.9 years (SD = 2.5, median = 9.5 years). Sixty-two patients (41.3%) died prior to the final follow-up period (January 1, 2019), with a median age at death of 78 years (range: [51, 97], mean = 77.1, SD = 9.0).

Among the exposed group, the median duration of exposure was 10 years (range: [3, 50], mean = 13.1, SD = 11.2). There were no differences between the occupational pesticide cohort and the control group in regard to age, sex, disease duration, Hoehn & Yahr score, medical comorbidities (according to the Charlson Comorbidity Index), and symptom onset [Table 1]. Pesticide exposure had a positive correlation with total UPDRS motor score, and this association remained significant when controlling for disease duration [Additional file 1].

**Table 1:** Clinical and socioeconomic data from PD patients with and without occupational pesticide exposure

Variable	All Patients	Occupational pesticide exposure?		*p* value^a^
		Yes	No	
Baseline characteristics	150	20	130	
Female sex (*n*, %)	81 (54%)	9 (45%)	72 (55.4%)	0.53
Age at study enrollment (in years, mean, SD)	64.4 (11.7)	63.2 (11.7)	64.6 (11.8)	0.61
Lost to follow up (*n*, %)	22 (14.7%)	2 (10%)	20 (15.4%)	0.77
Disease status				
Age at symptom onset (mean, SD)	56.5 (12.1)	54.2 (10.9)	56.8 (12.3)	0.34
Disease duration at study onset	7.9 (5.2)	9.0 (6.6)	7.8 (5.0)	0.45
Symptom onset before 50 years (*n*, %)	43 (28.7%)	9 (45%)	34 (26.2%)	0.14
Hoehn & Yahr Scale (mean, SD)	2.5 (0.8)	2.7 (0.9)	2.5 (0.8)	0.36
Total UPDRS score	51.6 (23.5)	67.0 (28.9)	49.5 (22.0)	***0.05***
Levodopa equivalent daily dose, mg (median, IQR)	750 [509, 1074]	775 [594, 1206]	750 [500, 1067]	0.67
Charlson Comorbidity Index	2 [1, 3]	2 [1, 3]	2 [1, 3]	0.49
Exposure history				
Any smoking history (*n*, %)	41 (27.3%)	6 (30%)	35 (26.9%)	0.79
Medium smoking history (10–30 pack-years)	14 (9.3%)	3 (15%)	11 (8.5%)	0.40
Heavy smoking history (> 30 pack years)	13 (8.7%)	1 (5%)	12 (9.2%)	1.0
Caffeine intake (at least 80 mg/day for 10 years)	92 (61.3%)	14 (70%)	78 (60%)	0.47
Socioeconomic status				
Patients in this subset (*n*)	105	14	91	
Education (fewer than 9 years, *n*, %)	25 (23.8%)	3 (22.2%)	22 (25%)	1.0
Race (white)	91.4%	94.4%	91.7%	1.0
Private health insurance coverage	17.1%	14.3%	17.6%	1.0
Historical monthly income < minimum wage (when fully employed)	9.5%	0%	17.6%	0.12
History of working predominately in agriculture	9.5%	21.4%	7.7%	0.13

^a^For dichotomous variables, *p*-value obtained from χ2 testing, or Fisher’s exact test for analyses with any values < 10. For continuous variables with normal distribution, *p*-value obtained from Welch’s two sample t-test (two-tailed). For continuous variables with non-normal distribution (as indicated by Shapiro-Wilk normality test), Wilcoxon signed rank test applied

Socioeconomic information was available for a subset of 105 patients, including 14 patients with occupational pesticide exposure [Table 1]. Eleven (78.6%) patients with a history of occupational pesticide exposure reported predominately working in non-agricultural occupations for a majority of their working life. A total of 17.6% of the control patients reported historically earning less than minimum wage when employed, compared to none of the patients with occupational pesticide exposure (*p* = 0.12). The pesticide exposure and control groups were similar in terms of race, private health insurance coverage, and educational attainment.

**Fig. 1:**
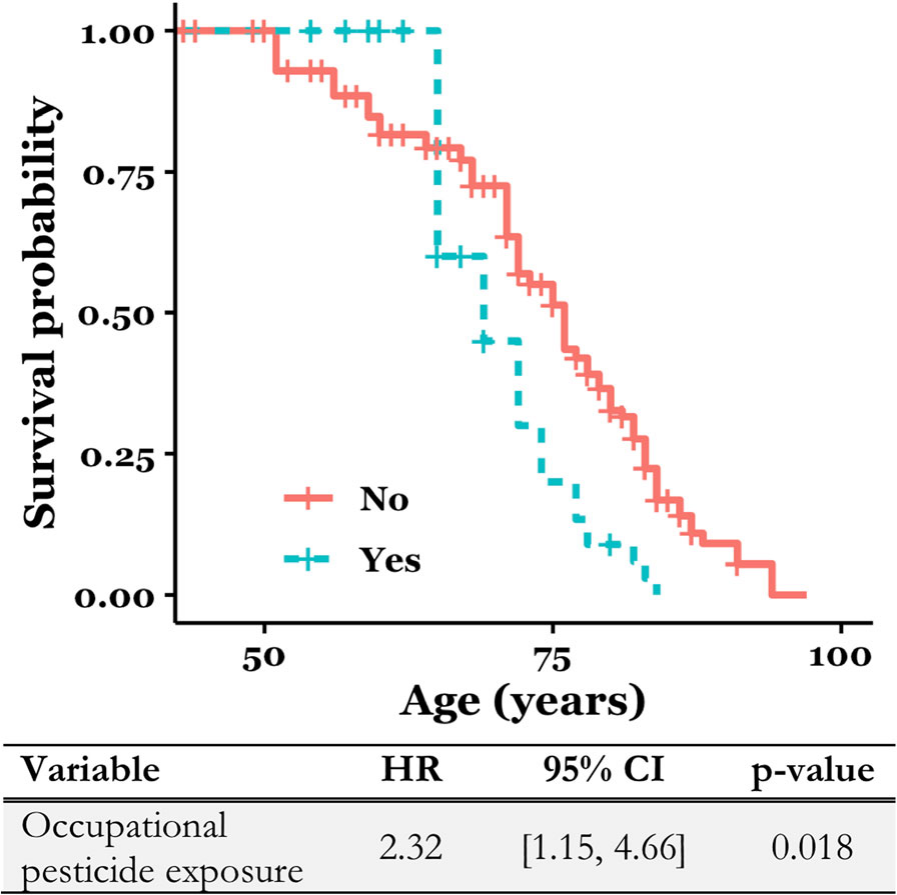
Survival curve comparing patients with (*n* = 20) and without (*n* = 130) self-reported occupational pesticide exposure (*n* = 150, *p*-value from log-rank test)

The survival curve of the occupational pesticide cohort was significantly different than the control group (log-rank test, *p* = 0.02). Patients with occupational pesticide exposure had a hazard of death two times as high as their unexposed PD counterparts (HR = 2.32, 95% CI [1.15, 4.66], *p* = 0.018) [Fig. 1]. The median survival was 76 years for the unexposed cohort versus 69 years for the exposed cohort. Although the survival curves crossed during the survival analysis due to early censoring of multiple subjects in the occupational pesticides cohort, the Schoenfeld residual test indicated that the proportional hazards assumption was not violated.

**Fig. 2:**
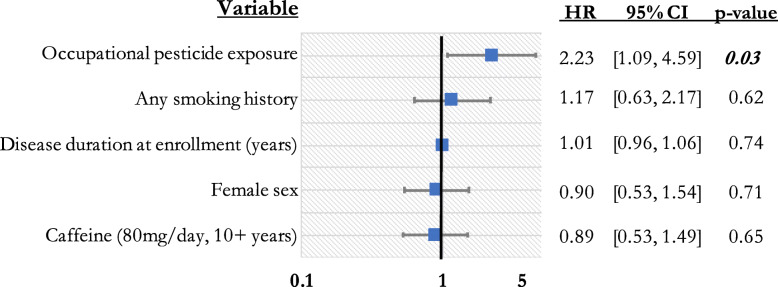
Impact of occupational pesticide exposure on mortality when controlling for smoking, caffeine intake, sex, and age (Multivariable cox proportional hazards model; *n* = 150, concordance = 0.60, SE = 0.04. Global Schoenfeld residual is *p* > 0.05))

Figure 2 displays the results of a cox proportional hazards regression that incorporated occupational pesticide exposure, sex, any smoking history, caffeine intake history (at least 80 mg/day for 10 or more years), and disease duration at enrollment. After adjusting for these exposure-related variables, occupational pesticide history was associated with a significantly elevated mortality rate (HR = 2.23, 95% CI [1.09, 4.59], *p* = 0.03) [Fig. 2]. A history of smoking, caffeine intake, and sex were not significant in this model.

**Fig. 3:**
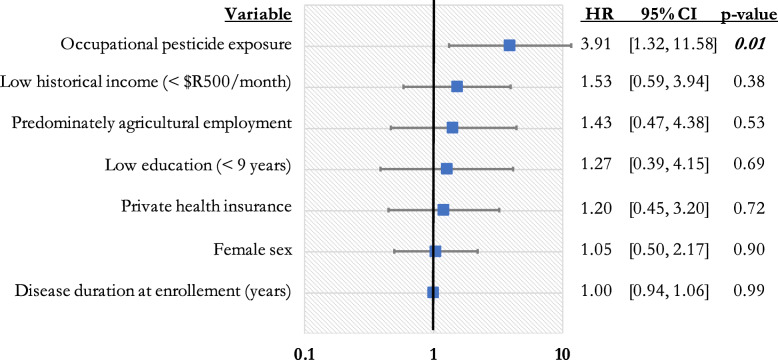
Impact of occupational pesticide exposure on mortality when controlling for sex, age, and socioeconomic status - employment history, average historical monthly income (when employed), insurance status, race, and education level (Multivariable cox proportional hazards model; *n* = 105, concordance = 0.60, se = 0.06 Global Schoenfeld residual is *p* > 0.05))

In addition, the relationship between several socioeconomic variables, sex, occupational pesticide exposure, and mortality was examined in a separate analysis. Similar to the prior regression, patients who reported occupational pesticide exposure had a higher mortality rate (HR = 3.91, 95% CI [1.32, 11.58], *p* = 0.01) [Fig. 3]. None of the socioeconomic covariables (low historical income, predominately agricultural employment, low education, private insurance coverage) were significant in this regression analysis.

Finally, the post-hoc analysis in Table 2 demonstrates a dose-dependent relationship between occupational pesticide exposure and the mortality rate. Patients with 10 or more years of occupational pesticide exposure had a significantly elevated mortality rate (HR = 2.81, 95% CI [1.17, 6.73], *p* = 0.02), in contrast to patients with fewer than 10 years of exposure [Table 2].

**Table 2:** Post-hoc analysis for dose-dependent effect of occupational pesticide exposure

Variable	Hazard Ratio	95% Confidence Interval	*p* Value
10 or more years of pesticide exposure	2.81	[1.17, 6.74]	*0.02*
Fewer than 10 years	1.84	[0.66, 5.17]	*0.25*

## Discussion

This is the first study performed in the LMIC/Latin American/Brazilian context of the relationship between occupational pesticide exposure and PD mortality. The existing literature regarding the risk of premature mortality among PD patients is equivocal – although multiple studies have indirectly linked pesticide exposure to an increased numerical rate of mortality, not all of these results have reached the threshold of statistical significance [14, 16, 17]. Among patients with PD, Caballero et al. demonstrated that individuals exposed to land-use associated with glyphosate in Washington state had an increased risk of premature mortality (OR = 1.33, 95%CI: [1.06, 1.67]), though this was not significant for those exposed to land-use associated with all pesticides [12]. Similarly, although pesticide use was associated with a higher hazard ratio (HR) among PD patients in the Netherlands, this difference was not significant (95%CI: [0.86, 1.88]) [15]. On the other hand, Ritz et al. demonstrated that there was an increase in PD-related mortality among patients who lived in Californian counties linked to higher levels of pesticide use [16]. In the present study, findings suggest that among a cohort of 150 patients with idiopathic PD in Southern Brazil, occupational pesticide exposure was associated with 2–4 times increased all-cause mortality rate. This association was significant in the crude (unadjusted) analysis as well as in adjusted analyses controlling for other exposure-related factors and socioeconomic factors.

According to Yan et al., there appears to be a dose-response relationship between duration of pesticide exposure and PD risk (5 year exposure OR = 1.05, 95% CI: [1.02–1.09]; 10 year exposure OR = 1.11, 95% CI: [1.05–1.18]) [23]. In addition, high organophosphate exposure is associated with a faster progression of motor and cognitive symptoms during a 7.5-year follow up period [3]. The findings presented in the present study contribute to this evidence by demonstrating that the occupational pesticide exposure is associated with an increased all-cause mortality rate among patients with PD. Furthermore, our dose-response analysis demonstrated that patients with 10 or more years of occupational pesticide exposure may have been driving the mortality difference in this study.

In the present study, the association between occupational exposure to pesticides and all-cause mortality among PD patients effect was significant even when controlling for socioeconomic contributors to lower life expectancy. Low educational attainment has been consistently linked to increased risk of death, with a recent study suggesting the effect on mortality may be comparable to smoking [24, 25]. Similarly poverty is a well-cited risk factor for chronic disease and premature mortality both in Brazil and across the globe [26, 27]. When specifically comparing socioeconomic status and PD mortality, Yang et al. found that low-income was associated to higher mortality rates of PD patients (HR = 1.12, 95% CI [1.09–1.15]) when compared to high-income patients. Interestingly, low-income controls had an even higher rate of mortality (HR = 1.35, 95% CI [1.35–1.36]), possibly because PD patients may have better control of different mortality risk factors for being followed-up in a hospital. Beard et al. identified higher mortality rates in the high-income population [28, 29]. In our study, the higher mortality rate associated with occupational pesticide exposure was still observed when accounting for current wealth (as measured by private health insurance coverage), a history of low income, and low educational attainment. None of the patients in the occupational pesticide exposure cohort in the present study reported earning less than minimum wage, suggesting that low socioeconomic status is not the driver for the higher rate of mortality among patients with occupational pesticide exposure among our cohort. Furthermore, although patients with low income our cohort had an elevated hazard ratio in the adjusted analysis (HR = 1.53, 95%CI: [0.59, 3.93], it is possible that this result was not statistically significant because there were few patients in this subset.

Given the inverse relationship between smoking and caffeine intake and the risk of developing PD, we sought to control for these factors in our analysis. Costa et al. found that caffeine has a dose dependent effect reducing the risk of PD with a relative risk (RR) of 0.76 per 300 mg of caffeine (95% CI: [0.72–0.80]) [30], to further support other studies linking caffeine intake to a reduced risk of developing PD [31–33]. This effect is seen especially in men [34] and includes various forms of caffeinated beverages. In addition, among a prospective cohort of 360 PD patients, coffee intake was found to be a protective factor against disease progression and mortality (HR = 0.47, 95%CI: [0.32, 0.69]) [35]. Smoking also has a significant protective effect with a RR of developing PD as low as 0.4 for a higher and longer history of intake [36]. In the present study, when controlling for these well-documented protective factors, occupational pesticide exposure remained statistically significantly associated with a 4-times higher hazard of mortality as compared to patients without occupational exposure to pesticides. Although caffeine intake and female sex were associated with lower hazard ratios, these differences were not statistically significant in the present study.

Interestingly, the majority of patients who reported occupational pesticide exposure did not report working predominately in agricultural jobs for the majority of their working life. In other words, since we only recorded the longest held occupation for each patient, most patients with fewer than 25 years of occupational pesticide exposure went on to work in other, non-agricultural professions for the majority of their working life. This suggests that a remote history of occupational pesticide exposure is associated with an increased risk of mortality in patients with PD, even among those who subsequently work in non-agricultural professions. Furthermore, the results from the multivariable cox proportional hazards model in Fig. 3 also support the notion that the pesticide exposure is truly driving this mortality difference, rather than other factors related to agricultural professions such as prolonged sun exposure or manual labor.

Unfortunately, most of patients in this study were unable to recall the specific agents that they were exposed to, limiting our ability to understand the potential variations in risk associated with different agrichemicals. Agricultural dependence on organophosphates and other pesticides continues to grow, creating an urgent need to better characterize the neurologic consequences of specific agents [2].

A notable limitation of this study is that we were unable to separate Parkinson-specific mortality from all-cause mortality due to the nature of the medical records and national obituary records. Therefore, our study was unable to conclusively determine if the link between pesticide exposure and mortality is truly due to faster progression of PD, even though we found that the motor UPDRS score was higher in the pesticide group when controlling for disease duration. Although it is possible that the increased risk of death is attributable to other exposure-related medical conditions, the baseline health status at the time of enrollment was comparable between the two cohorts, as measured by the Charlson Comorbidity Index. This further supports our findings regarding the association between pesticide exposure and higher mortality rate.

Another limitation is the various enrollment dates (2008–2013) for the cohort, which could have induced time-based differences in the level of care received by the patients. However, statistical tests implemented to check for this possibility (Schoenfeld residuals of each of the univariable analyses and the multivariable cox proportional hazard regressions) were not significant, indicating that there was not a measurable time-related component to the variables used in this analysis. Because patients are of advanced age and were asked to report on occupational exposure throughout their lifetime, the possibility of recall bias cannot be excluded. However, because all patients interviewed had the same clinical condition and the questions about exposure were asked before the mortality outcomes were ascertained, we believe the possibility for recall bias is minimal.

Among this cohort of moderately-advanced PD patients, individuals with more severe variants or rapid disease progression may not have been captured in this analysis since they may have died before enrollment. Patients with occupational pesticide exposure were younger at the time of enrollment, and a larger proportion of the exposed patients were considered to be “early onset” PD patients (onset before the age of 50, 45% vs 26.2, *p* = 0.14) though this difference was not statistically significant. Given that patients with early onset PD tend to have longer disease durations, but die at younger ages than other patients with PD [37], future studies would benefit from following patients from symptom onset or diagnosis in order to fully understand the impact of these socioeconomic and exposure related factors in a more prognostically meaningful way.

## Conclusion

In this prospective cohort study, we found an increased all-cause mortality rate in PD patients with occupational exposure to pesticides. This rate was controlled for sex, smoking, caffeine intake, and socioeconomic status. Even though the study does not account for specific pesticides, paraquat is still permitted in Brazil and glyphosate is widely used in many plantations including soybeans, which are one of the most important agricultural exports in the country. In this context of increasing prevalence of exposure by extremely toxic, recently approved new pesticides, this information is highly relevant. More studies are needed to further analyze this topic with longer follow-up periods, more detailed exposure information, and more specific causes of mortality. This is especially important in the Brazilian market, and perhaps in other developing countries, where new pesticides continue to be introduced without the corresponding research output necessary to understand the impact on human health [18].

## Supplementary information

**Additional file 1.** Linear regression demonstrating the relationship between occupational pesticide exposure and UPDRS-III score, when controlling for disease durations.

## Data Availability

The datasets used and/or analysed during the current study are available from the corresponding author on reasonable request.

## Abbreviations

PD: Parkinson’s disease
MPP + 1: Methyl-4-phenylpyridinium
MPTP: 1-methyl-4-phenyl-1,2,3,6-tetrahydropyridine
HCPA: Hospital de Clínicas de Porto Alegre
UPDRS: Unified Parkinson’s Disease Rating Scale
