# Supplementary material for: Occupational pesticide exposure and the risk of death in patients with Parkinson’s disease: an observational study in southern Brazil
Source: Environ Health. 2020 Jun 17;19:68. doi: 10.1186/s12940-020-00624-8 (PMC7298782; doi:10.1186/s12940-020-00624-8)
Supplement: Supplementary file 1 — Additional file 1. Linear regression demonstrating the relationship between occupational pesticide exposure and UPDRS-III score, when controlling for disease durations. [file 12940_2020_624_MOESM1_ESM.docx]

| Variable | Coefficient (β) | Standard Error | p Value | 95% Confidence Interval |
| --- | --- | --- | --- | --- |
| Intercept | 23.39 | 2.49 |  |  |
| Occupational pesticide exposure | 9.93 | 3.94 | ***0.01*** | ***(2.14, 17.71)*** |
| Disease duration at enrollment, in years | 0.84 | 0.26 | ***<0.01*** | ***(0.34, 1.35)*** |

**Additional File 1:** Linear regression demonstrating the relationship between occupational pesticide exposure and UPDRS-III score, when controlling for disease duration
